# Establishment of a co-culture system using *Escherichia coli* and *Pichia pastoris* (*Komagataella phaffii*) for valuable alkaloid production

**DOI:** 10.1186/s12934-021-01687-z

**Published:** 2021-10-18

**Authors:** Miya Urui, Yasuyuki Yamada, Yoshito Ikeda, Akira Nakagawa, Fumihiko Sato, Hiromichi Minami, Nobukazu Shitan

**Affiliations:** 1 Laboratory of Medicinal Cell Biology, Kobe Pharmaceutical University, Motoyamakita-machi, Higashinada-ku, Kobe, 658-8558 Japan; 2 Research Institute for Bioresources and Biotechnology, Ishikawa Prefectural University, Nonoichi-shi, Ishikawa 921-8836 Japan; 3 Department of Plant Gene and Totipotency, Division of Integrated Life Science, Graduate School of Biostudies, Kyoto University, Kyoto, 606-8502 Japan; 4 Graduate School of Science, Osaka Prefecture University, Sakai, 599-8531 Japan

**Keywords:** Benzylisoquinoline alkaloid, Stylopine, Reticuline, *Escherichia coli*, *Pichia pastoris*, Metabolic engineering, Co-culture system, Medium

## Abstract

**Background:**

Plants produce a variety of specialized metabolites, many of which are used in pharmaceutical industries as raw materials. However, certain metabolites may be produced at markedly low concentrations in plants. This problem has been overcome through metabolic engineering in recent years, and the production of valuable plant compounds using microorganisms such as *Escherichia coli* or yeast cells has been realized. However, the development of complicated pathways in a single cell remains challenging. Additionally, microbial cells may experience toxicity from the bioactive compounds produced or negative feedback effects exerted on their biosynthetic enzymes. Thus, co-culture systems, such as those of *E. coli*–*E. coli* and *E. coli*-*Saccharomyces cerevisiae*, have been developed, and increased production of certain compounds has been achieved. Recently, a co-culture system of *Pichia pastoris* (*Komagataella phaffii*) has gained considerable attention due to its potential utility in increased production of valuable compounds. However, its co-culture with other organisms such as *E. coli*, which produce important intermediates at high concentrations, has not been reported.

**Results:**

Here, we present a novel co-culture platform for *E. coli* and *P. pastoris*. Upstream *E. coli* cells produced reticuline from a simple carbon source, and the downstream *P. pastoris* cells produced stylopine from reticuline. We investigated the effect of four media commonly used for growth and production of *P. pastoris*, and found that buffered methanol-complex medium (BMMY) was suitable for *P. pastoris* cells. Reticuline-producing *E. coli* cells also showed better growth and reticuline production in BMMY medium than that in LB medium. De novo production of the final product, stylopine from a simple carbon source, glycerol, was successful upon co-culture of both strains in BMMY medium. Further analysis of the initial inoculation ratio showed that a higher ratio of *E. coli* cells compared to *P. pastoris* cells led to higher production of stylopine.

**Conclusions:**

This is the first report of co-culture system established with engineered *E. coli* and *P. pastoris* for the de novo production of valuable compounds. The co-culture system established herein would be useful for increased production of heterologous biosynthesis of complex specialized plant metabolites.

**Supplementary Information:**

The online version contains supplementary material available at 10.1186/s12934-021-01687-z.

## Background

Specialized metabolites produced by plants, also known as secondary metabolites, exhibit diverse chemical structures and biological activities. Several metabolites have been used as drugs, such as morphine for the development of analgesics, artemisinin used as an anti-malarial drug, and vinblastine used as an anti-cancer drug [1]. Though more stable industrial production and supply of such useful compounds is necessary, many of the metabolites are unsuitable for chemical synthesis owing to their complex chemical structures and laborious extraction procedures from plants. Owing to the decrease in plant resources and their low concentrations in plant cells, the stable supply of some such compounds may be challenging in the future. To solve these problems, biosynthetic enzymes have been investigated, with their corresponding genes identified and isolated. In recent years, metabolic engineering, a process in which genes for biosynthetic enzymes are introduced into microorganisms such as *Escherichia coli* or *Saccharomyces cerevisiae*, leading to successful production of valuable compounds has been reported [2, 3]. Few examples are as follows: the production of thebaine, an important opiate, by *E. coli* [4] and *S. cerevisiae* [5]; the production of tropane alkaloids, that act as neurotransmitter inhibitors, by *S. cerevisiae* [6]; and the production of resveratrol, a stilbene with potential health-promoting benefits, by *E. coli* and *S. cerevisiae* [7].

**Fig. 1:**
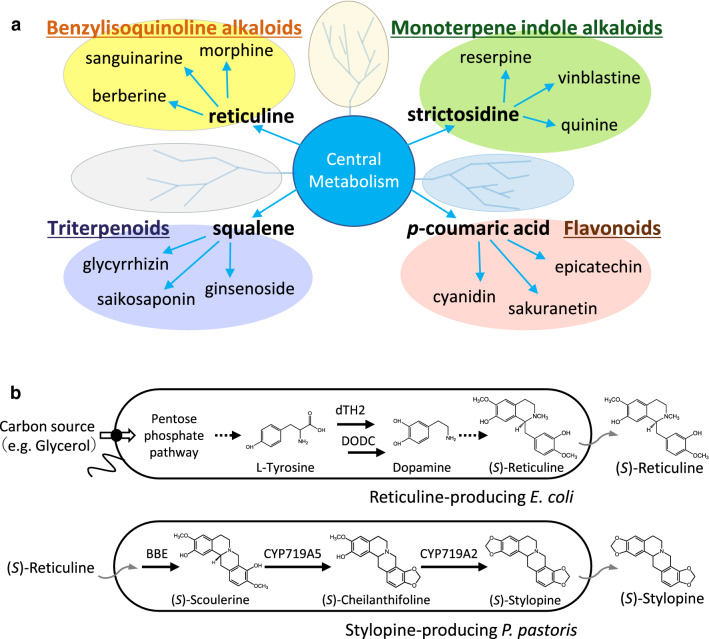
Metabolites biosynthesized in plants (**a**) and cells used for de novo stylopine production (**b**). **a** Various specialized metabolites are produced from common intermediates like reticuline, strictosidine, *p*-coumaric acid, squalene, and others. These intermediates are derived from central metabolism. **b** Reticuline-producing *E. coli* cells (AN2014 strain) produce (*S*)-reticuline using simple carbon sources such as glucose or glycerol, via three engineered pathways, namely (1) an l-tyrosine-overproducing pathway, (2) a dopamine and 3,4-dihydroxyphenylacetaldehyde (3,4-DHPAA) production pathway from l-tyrosine, and (3) a reticuline-producing pathway from dopamine. Stylopine-producing *P. pastoris* (B52 strain) produce (*S*)-stylopine from (*S*)-reticuline via three steps catalyzed by berberine bridge enzyme (BBE), cheilanthifoline synthase (CYP719A5), and stylopine synthase (CYP719A2)

Contrary to successful reports, there are a few compounds that cannot be biosynthesized at high concentrations in microorganisms, probably due to the cytotoxicity of substrates added to the medium or end-products, or the exertion of negative feedback on the biosynthetic enzymes. Additionally, the construction of the entire biosynthetic pathway, including the selection of the most suitable host cell, introduction of multiple biosynthetic genes, and examination of enzyme expression conditions, in a single cell, involves considerable efforts and is time-consuming. To circumvent these problems, we focused on the fact that several specialized metabolites are derived from common intermediates. Various benzylisoquinoline alkaloids (BIAs) are derived from reticuline, and different monoterpeneindole alkaloids originate from strictosidine; furthermore, *p*-coumaric acid helps in the generation of phenylpropanoids including flavonoids, and triterpenoids are derived from squalene [8] (Fig. 1a). Therefore, establishment of a co-culture system of microorganisms, each possessing complementary or split pathways, may be a useful strategy for the efficient production of valuable compounds [9–11]. In recent years, several reports have demonstrated the co-culture system to be a powerful tool for large-scale production of various compounds. Notable examples include biosynthesis of sakuranetin [12] or anthocyanins [13] using co-culture of *E. coli*–*E. coli* (strains engineered with different genes), and biosynthesis of resveratrol [7], or magnoflorine [14] using a co-culture of *E. coli* and *S. cerevisiae*. Recently, a co-culture system with *Corynebacterium glutamicum* and *E. coli* has also been reported for the production of lysine-derived metabolites, cadaverine, and l-pipecolic acid [15].

Novel co-culture systems could be employed to produce compounds hitherto not investigated or could not be generated through the already known combinatorial systems. *Pichia pastoris* (*Komagataella phaffii*) is a methylotrophic yeast that has been used for industrial scale production of recombinant proteins. *P. pastoris* has also been used for the production of valuable compounds in recent years owing to an increased expression of biosynthetic enzymes [16–18]. Compounds successfully produced using *P. pastoris* include lovastatin [19], dammarenediol-II [20], nootkatone [21], ambrein [22], stylopine [23], amongst others. The advantage of using *P. pastoris* as a host cell is that certain enzymes that are not functional or exhibit low activity in other organisms present with a high conversion rate in this cell. For example, cytochrome P450 enzyme CYP719A5 derived from *Eschscholzia californica*, which catalyzes the conversion of scoulerine to cheilanthifoline, showed a higher conversion rate in *P. pastoris* cells (70%) compared to *S. cerevisiae* cells (20%) [23]. The construction of the entire biosynthetic pathway, however, is challenging in a single *P. pastoris* cell. Therefore, a splitting pathway was proposed and a co-culture of *P. pastoris*–*P. pastoris* was performed with different cells synthesizing different enzymes [19, 23]. However, knowledge of co-culture systems of *P. pastoris* with other organisms is limited; particularly, its co-culture method with *E. coli* has not been established. *E. coli* is a standard microorganism used for industrial-scale production of different compounds [24, 25], and high production of certain common intermediates for specialized metabolites, such as reticuline [26], *p*-coumaric acid [7], and squalene [27] by *E. coli* cells has been reported. However, *E. coli* is a prokaryotic cell and lacks subcellular organelles essential for the expression and function of certain enzymes such as cytochrome P450. Therefore, in such cases where further modification of the basic structure of a metabolite through enzymes such as P450 is required, use of eukaryotic cells is more suitable. As described above, *P. pastoris* shows high protein expression and a higher conversion rate than *S. cerevisiae* in a few cases. Therefore, a co-culture system for *P. pastoris* and *E. coli* may be useful for the increased production of valuable compounds.

In this study, we established a co-culture system for *E. coli* and *P. pastoris*. Four vectors harboring 14 genes were introduced in *E. coli*, the upstream strain, to enable production of (*S*)-reticuline, an important intermediate for various BIAs, using a simple carbon source such as glucose or glycerol [26] (Fig. 1b) (Additional file 1: Table S1). The downstream strain, *P. pastoris*, which was established via the introduction of three *E. californica* genes, namely berberine bridge enzyme (BBE), cheilanthifoline synthase (CYP719A5), and stylopine synthase (CYP719A2), into the genome, resulted in the production of (*S*)-stylopine, a potential anti-inflammatory compound [28], from (*S*)-reticuline [23] (Fig. 1b) (Additional file 1: Table S1). Here, we first investigated the effect of several media on *P. pastoris* cell growth and the biosynthesis of stylopine. We then determined the optimum medium and initial inoculation ratios for co-culture. We report the first establishment of *E. coli*-*P. pastoris* co-culture system, which can be used to produce (*S*)-stylopine from glycerol. This platform would be helpful for conducting combinatorial biotransformation of a variety of useful components (Fig. 1).

## Results and discussion

### Appropriate medium for stylopine production in *P. pastoris*

**Fig. 2:**
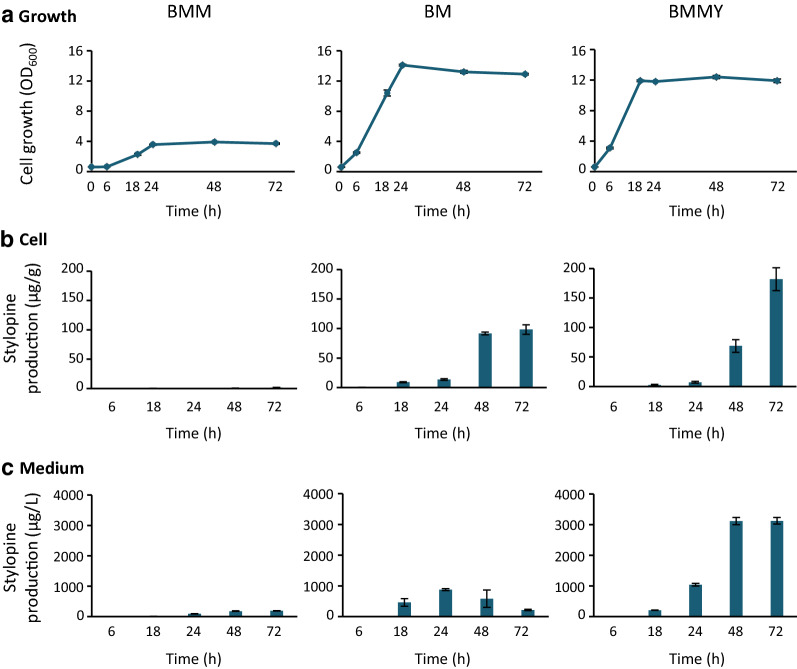
Growth of *P. pastoris* and (*S*)-stylopine production from (*S*)-reticuline in various media. Growth was evaluated by measuring the optical density at 600 nm (**a**), and the time-dependent production of (*S*)-stylopine in *P. pastoris* cells (**b**) and the medium (**c**) was determined. *P. pastoris* cells were cultured in each medium containing 100 µM (*S*)*-*reticuline, and sampled at the times indicated. Results indicate mean ± standard deviation of triplicate experiments

For *P. pastoris* culture, few basic media (Table 1), such as minimal methanol (MM), buffered minimal methanol (BMM), and buffered methanol-complex medium (BMMY), developed by Invitrogen Co. have been commonly used. However, knowledge about the effects of these media on the production of stylopine is limited. Therefore, we tested these three media and BM medium, the use of has been reported in a previous study [29], to determine a suitable medium that could be used for cell growth and stylopine production (Table 1). Stylopine-producing *P. pastoris* cells, pre-cultured in YPD medium, were inoculated at OD_600_ = 0.6, in each medium containing reticuline as a substrate, and incubated at 30 °C under shaking conditions (250 rpm). The cell growth and stylopine biosynthesis were monitored. Since stylopine production was not observed in MM medium (Additional file 2: Fig. S1), the data in other media are shown (Fig. 2). Cells cultured in other media exhibited exponential growth up to 18–24 h, and then entered the stationary phase (Fig. 2a). Early induction of the stationary phase and limited cell growth was observed in the BMM medium, compared to other media. It should be noted that stylopine concentrations in cells and medium differed significantly between media (Fig. 2b, c). Although stylopine was present in the BMM medium, which is a buffered MM medium (Table 1), its production rate was the lowest compared to other media. It could be inferred that stylopine production might be influenced by the pH of the medium. In the BM medium, stylopine was produced at a higher concentration and was predominantly accumulated in cells after 48 h. In contrast, in the BMMY medium, considerable proportion of the stylopine produced was present in the medium. At 72 h, stylopine content in the BMMY medium was 14.3-fold higher than that in BM medium (3125 µg/L in BMMY and 218 µg/L in BM medium). These results indicate that the type of medium used exerts a significant effect on stylopine production and its efflux into the medium. Considering that the efficient recovery of end-product was possible from the medium, without the extraction from cells, we considered the BMMY medium to be more appropriate for stylopine production and used this medium for further analysis.

**Table 1:** Composition of medium used in this study

Component	MM	BMM	BM	BMMY	LB
Methanol	0.5%	0.5%	1%	0.5%	–
YNB (Yeast Nitrogen Base)	1.34%	1.34%	–	1.34%	–
Biotin	4 × 10^−5^%	4 × 10^−5^%	–	4 × 10^−5^%	–
Potassium phosphate buffer, pH 6.0	–	100 mM	–	100 mM	–
Yeast extract	–	–	0.5%	1%	0.5%
Peptone	–	–	–	2%	–
Tryptone	–	–	–	–	1%
NaCl	–	–	–	–	1%
5 N NaOH	–	–	–	–	pH 7.0

MM: minimal methanol; BMM: buffered minimal methanol; BMMY: buffered methanol-complex medium

### Reticuline production using *E. coli* in the BMMY medium

**Fig. 3:**
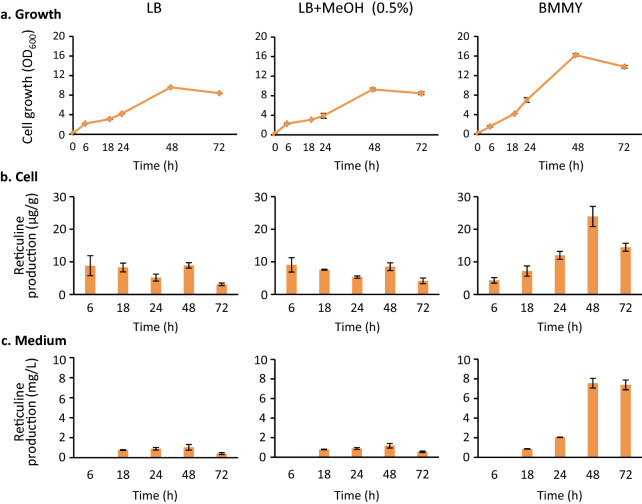
Growth of *E. coli* and (*S*)-reticuline production from glycerol. Growth was evaluated by measuring the optical density at 600 nm (**a**), and the time-dependent production of (*S*)-reticuline in *E. coli* cells (**b**) and the medium (**c**) was determined. *E. coli* cells were cultured in LB, LB containing MeOH (0.5%) or the BMMY medium. These media contained IPTG (0.1 mM), glycerol (5 g/L), and appropriate antibiotics. Cells and media were sampled at the times indicated. Results indicate mean ± standard deviation of triplicate experiments

Next, we examined the effect of MeOH, which was added to the BMMY medium to induce protein expression in *P. pastoris*, on the growth and reticuline production in reticuline-producing *E. coli* cells. *E. coli* cells were pre-cultured in Luria Bertani (LB) medium, and then inoculated in either LB medium, LB medium containing MeOH, or BMMY medium, and cultured under shaking conditions (250 rpm) at 30 °C. All media contained isopropyl β-d-thiogalactopyranoside (IPTG) (0.1 mM) to induce the biosynthetic enzymes for reticuline in *E. coli* cells. In LB medium, the addition of 0.5% MeOH exerted negligible effect on cell growth and reticuline production (Fig. 3). Surprisingly, better cell growth was observed in the BMMY medium, compared to the LB medium. Additionally, reticuline was efficiently produced in the BMMY medium. After incubation for a duration of 24 h, the cellular reticuline content in BMMY was 2 to 3.5 times higher and its concentration in BMMY medium was 2 to 13 times higher than that observed in LB medium. At 72 h, the reticuline content in the BMMY medium was found to be 7.4 mg/L, which was 13-fold higher than that observed in the LB medium containing MeOH (0.56 mg/L). Reticuline is secreted into the BMMY medium and this is desirable for efficient transfer of the biosynthetic intermediate in the co-culture system. Therefore, we selected this medium for use in the co-culture system.

### *E. coli*-*P. pastoris* co-culture system for stylopine production

**Fig. 4:**
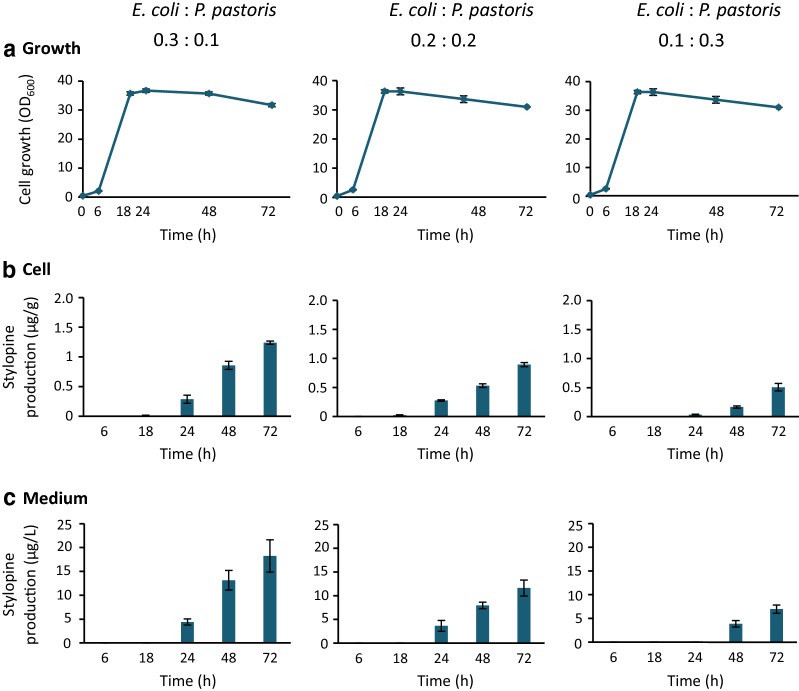
De novo (*S*)-stylopine production from glycerol using the co-culture of *E. coli* and *P. pastoris*. Reticuline-producing *E. coli* and stylopine-producing *P. pastoris* were pre-cultured in LB medium containing antibiotics and YPD medium, respectively. The cultures were inoculated, at the initial ratio indicated, into the BMMY medium containing IPTG (0.1 mM), glycerol (5 g/L), and antibiotics. Growth was estimated by measuring the optical density at 600 nm (**a**), and the time-dependent production of (*S*)-stylopine in cells (**b**) and the medium (**c**) was determined. Results indicate mean ± standard deviation of triplicate experiments

We investigated whether the co-culture of reticuline-producing *E. coli* and stylopine-producing *P. pastoris* could lead to the de novo production of stylopine from a simple carbon source (Fig. 1b). *E. coli* and *P. pastoris* cells were pre-cultured in LB and YPD media, respectively. Both cells were co-cultured in the BMMY medium containing IPTG, MeOH, and glycerol as a simple carbon source. This culture system showed successful in de novo production of stylopine from glycerol. Therefore, we further investigated the effect of initial inoculation ratio on stylopine production. *E. coli* and *P. pastoris* cells inoculated at ratios of 0.3:0.1, 0.2:0.2, and 0.1:0.3, acquired at OD_600_, showed similar exponential cell growth up to 18 h, after which they entered the stationary phase (Fig. 4a). Stylopine production was observed in all cases and was the highest in the 0.3:0.1 ratio in both the cells and the medium (Fig. 4b and c); higher the ratio of *E. coli* cells, higher the rate of stylopine production. At 72 h, the stylopine content in the medium was found to be approximately 20 µg/L. These results indicate that the upstream *E. coli* strain is the rate-limiting factor for stylopine production in this co-culture system. Consistent with this hypothesis, reticuline, an important intermediate produced by *E. coli* cells, was undetectable at most time points in both the cells and the medium (Additional file 2: Fig. S2). The growth of *E. coli* cells in co-culture (Additional file 2: Fig. S3) was not as well as that in a single culture (Fig. 3), therefore, the higher ratio of *E. coli* cells would be suitable for higher production of stylopine.

As illustrated in Fig. 3, reticuline production in the *E. coli* cells was observed from 6 h, while the reticuline secreted into the medium increased after 18 h. In contrast, the induction of biosynthetic enzymes in *P. pastoris* cells seemed to require 18 h or more, since stylopine production was observed after 18 h even when reticuline was added to the medium at the beginning of the experiment (Fig. 2). Similar conditions would be required for induction of biosynthetic enzymes and biosynthesis in *P. pastoris* present in the co-culture system, since reticuline was observed in the cells and the medium only at 6 h (Additional file 2: Fig. S2). For a duration of up to 18 h, the downstream *P. pastoris* strain might be the rate-limiting factor for stylopine production. The earlier induction of biosynthesis enzymes in *P. pastoris* during pre-culture might accelerate the production of stylopine and relieve this limitation. In addition, successive feeding of the substrate, glycerol, in this case, might enhance the productivity, as previously reported for *P. pastoris* [23].

In this study, only stylopine production was investigated, using reticuline as a common intermediate. However, various compounds such as thebaine and resveratrol have been produced from *E. coli*–*E. coli* or *E. coli*–*S. cerevisiae* co-culture, using some common intermediates such as reticuline and *p*-coumaric acid [7, 12–14]. In addition, in the co-culture of *P. pastoris*–*P. pastoris*, the production of lovastatin and stylopine was reported [19, 23]. These suggest that *E. coli* and *P. pastoris* are relatively able to efflux or influx the various intermediates. Therefore, the co-culture system established in this study would be applicable to the production of other valuable metabolites, through the other intermediates.

We selected a co-culture system of *E. coli* cells and *P. pastoris* cells. *E. coli* is suitable for the high production of some intermediates derived from central metabolites [30], i.e., reticuline and *p*-coumaric acid, and downstream *P. pastoris* is appropriate for further modification of intermediates using well expressed enzymes like BBE and P450. However, recently, higher production of reticuline was reported using *S. cerevisiae* [31]. In the future, co-cultivation of *S. cerevisiae* and *P. pastoris* might be useful. Since this is a co-culture of yeast cells, that is, *S. cerevisiae*-*P. pastoris*, optimization of the growth condition and productivity might be easier than co-culture of *E. coli*-*P. pastoris*. Production of diverse valuable metabolites will become possible through the application of various co-culture systems, including the co-culture system between *E. coli* and *P. pastoris* established in this study.

In a co-culture system, efficient transfer of biosynthetic intermediates between cell lines is also important. We showed that the expression of an alkaloid transporter, *Arabidopsis thaliana* DTX1, in reticuline-producing *E. coli* cells significantly enhanced reticuline production and its efflux into the medium [32]. Therefore, the use of this transporter-expressing *E. coli* in the present co-culture system may lead to enhanced production of stylopine. In a previous study, we have also shown that enhanced reticuline efflux into the medium releases the negative feedback on the biosynthetic enzymes such as methyltransferases, leading to the induction of reticuline-related biosynthesis pathways in the cells [32]. In the present co-culture, reticuline, released from *E. coli* cells, was quickly converted by *P. pastoris* cells and its concentration in the medium was low, which might have enhanced reticuline production by *E. coli* cells. Substrate uptake by *P. pastoris* cells is also important. It has been reported that expression of a purine permease, BUP1, which performs the uptake of the intermediates of BIA, in *S. cerevisiae* expressing thebaine biosynthetic enzymes, significantly improves thebaine production [33]. Since this transporter showed reticuline uptake activity, expression of this transporter in stylopine-producing *P. pastoris* might also lead to improvement of substrate transfer and productivity. Transport engineering may also contribute to the development of a co-culture system in the future.

## Conclusions

We successfully developed an *E. coli-P. pastoris* co-culture platform that enabled de novo production of a valuable alkaloid, stylopine. The BMMY medium is appropriate for production and secretion of compounds into the medium in both *E. coli* and *P. pastoris*. Metabolite production increased when the *E. coli* ratio was higher in the co-culture system. The results of this study are of considerable significance since *P. pastoris* is a novel microorganism used for the industrial production of pharmaceuticals [16–18]. This platform can potentially lead to a low-cost and stable supply of various valuable compounds.

## Methods

### Chemicals

(*S*)-Reticuline was synthesized and purified as per methods previously described [26]. (*S*)-Stylopine was prepared from coptisine chloride purchased from FujiFilm Wako Pure Chemical Corporation (Osaka, Japan); the preparation was achieved via chemical reduction with sodium borohydride.

### Reticuline-producing *Escherichia coli* and stylopine-producing yeast cells

Reticuline-producing *E. coli* cells (designated as the AN2104 strain) were generated by introducing four plasmids, for genes encoding reticuline biosynthetic enzymes, as per protocols described previously [32] (Additional file 1: Table S1). *P. pastoris* cells (designated as the B52 strain), containing three genes encoding biosynthetic enzymes (BBE, CYP719A5, and CYP719A2) and enabling the production of stylopine from reticuline, were also generated as per methods described previously [23] (Additional file 1: Table S1).

### Stylopine production from (*S*)-Reticuline by *P. pastoris* cells in different culture media

Stylopine-producing B52 cells were grown in the YPD medium (1% yeast extract, 2% peptone, and 2% dextrose) at 30 °C under shaking conditions (200 rpm) until the achievement of an OD_600_ of 3. The cells were then collected and resuspended in either MM (1.34% YNB, 4 × 10^−5^% biotin, 0.5% methanol), BMM (100 mM potassium phosphate, pH 6.0, 1.34% YNB, 4 × 10^−5^% biotin, 0.5% methanol), BM (0.5% yeast extract, 1% methanol), or BMMY (1% yeast extract, 2% peptone, 100 mM potassium phosphate, pH 6.0, 1.34% YNB, 4 × 10^−5^% biotin, 0.5% methanol) medium at an OD_600_ of 0.6. After supplementing with 100 µM (*S*)-reticuline, the cells were incubated at 30 °C under shaking conditions (250 rpm) and sampled at 6, 18, 24, 48, and 72 h along with the culture medium.

### Reticuline production from glycerol by *E. coli* cells in the BMMY medium

Reticuline-producing AN2104 cells were pre-cultured overnight at 30 °C under shaking conditions (200 rpm) in LB medium containing appropriate antibiotics (2 mg/L tetracycline [Nacalai Tesque, Kyoto, Japan], 80 mg/L ampicillin [Sigma-Aldrich, St. Louis, MO, USA], 100 mg/L spectinomycin [Nacalai Tesque], and 30 mg/L chloramphenicol [Nacalai Tesque]). The overnight culture was inoculated at OD_600_ = 0.2 in fresh LB medium containing appropriate antibiotics and the cells were grown for 2 h at 30 °C until the achievement of an OD_600_ of 0.6. The cells were then collected and resuspended in the LB, LB containing MeOH (0.5%) or BMMY medium, containing appropriate antibiotics, IPTG (0.1 mM), and glycerol (5 g/L) with an initial OD_600_ of 0.2, and were incubated at 30 °C under shaking conditions (250 rpm). The samples were harvested at 6, 18, 24, 48, and 72 h after induction.

### De novo production of stylopine from co-culture of *E. coli* and *P. pastoris* cells in the BMMY medium

Reticuline-producing AN2104 cells were pre-cultured in LB medium containing the appropriate antibiotics, as per methods described above. Stylopine-producing B52 cells were pre-cultured in YPD medium as per methods described above. Both types of pre-cultured cells were collected and resuspended in the BMMY medium containing 0.1 mM IPTG, appropriate antibiotics, and glycerol, at the initial concentration ratios of OD_600_ as indicated earlier. The cells were incubated at 30 °C under shaking conditions (250 rpm). The samples were harvested at 6, 18, 24, 48, and 72 h after induction.

### Metabolite analysis

All culture samples were centrifuged and separated into supernatants (medium) and pellets (cells). Trichloroacetate (2% final concentration) was added to the supernatant to precipitate the proteins, followed by centrifugation at 15,000×*g* for 20 min. The pellets were subjected to washing steps with ice water and incubated overnight with 40 µL/mg fresh weight (FW) (for *E. coli*) or 20 µL/mg FW (for *P. pastoris*) methanol containing 0.01 N HCl. These samples were then centrifuged at 15,000×*g* for 15 min, and the supernatants obtained thereafter were used for analysis.

All samples were filtered using 0.45 μm Cosmospin Filters (Nacalai Tesque), and analyzed by conducting UPLC-MS using the ACQUITY UPLC system with QDa mass detector (Waters Corp., Milford, MA, USA); mobile phase comprised 0.01% (v/v) acetic acid in water (solvent A) and 0.01% (v/v) acetic acid in acetonitrile (solvent B). Alkaloids were separated via gradient elution as follows: mobile phase was subjected to linear decrease from 95% A to 60% A in 9 min, following decrease from 60% A to 50% A in 3 min, and increase from 50% A to 95% A in 3 min; column, CORTECS UPLC C18 (1.6 μm, 2.1 × 100 mm; Waters Corp.) was used considering temperature of 40 °C with a flow rate of 0.3 mL/min.

The QDa conditions were set as follows: cone voltage, 15 V; capillary voltage, 0.8 kV; and source temperature, 600 °C. Reticuline (m/z = 330), and stylopine (m/z = 324) were detected using the single-ion recording (SIR) mode, and each peak was identified by conducting direct comparison with peaks corresponding to authentic standard chemicals. The content of each alkaloid was quantified using a standard curve.

## Supplementary Information

**Additional file 1: Table S1.** Plasmids used in this study.

**Additional file 2: Fig. S1.** Detection of (*S*)-stylopine in various media. Single-ion chromatogram of (*S*)-stylopine in *P. pastoris* cells grown in each medium and authentic standard. N.D.; not detected. **Fig. S2** (*S*)-Reticuline production in the co-culture of *E. coli* and *P. pastoris*. Cells were cultured as per methods described in the Fig. 4 legend. (*S*)-Reticuline in the cells (**a**) and medium (**b**) were detected and quantified. Results indicate mean ± standard deviation of triplicate experiments. **Fig. S3** Growth and cell density of *E. coli* and *P. pastoris* in co-culture system. An initial inoculation ratio of *E. coli and P. pastoris* cells was 0.3:0.1. Growth was evaluated by measuring the optical density at 600 nm (a). The number of *E. coli* cells (b) and *P. pastoris* cells (c) were counted using a bacteria counting chamber and a microscope. Results indicate mean ± standard deviation of technical triplicates.

## Data Availability

The datasets used and/or analyzed during the current study are available from the corresponding author on reasonable request.
