# Supplementary material for: Establishment of a co-culture system using Escherichia coli and Pichia pastoris (Komagataella phaffii) for valuable alkaloid production
Source: Microb Cell Fact. 2021 Oct 18;20:200. doi: 10.1186/s12934-021-01687-z (PMC8522034; doi:10.1186/s12934-021-01687-z)
Supplement: Supplementary file 1 — Additional file 1: Table S1. Plasmids used in this study. [file 12934_2021_1687_MOESM1_ESM.docx]

**Table S1. Plasmids used in this study**

| Plasmid | Description | Source |
| --- | --- | --- |
| pMW118tet | Tetracycline resistant, modified expression vector, pMW118 | Yamada et al., Metab Eng Commun., 2021 |
| pMW-TyrOE | *tyrA^fbr^*, *aroG^fbr^*, *tktA*, and *ppsA* in pMW118tet | Yamada et al., Metab Eng Commun., 2021 |
| pCDF-MPSdTH2op | *BsMtrAop*, *RnPTPSop*, *RnSPRop*, and *dTH2op* in pCDFPL | Matsumura et al., Sci Rep., 2018 |
| pET-NMop | *NCSop* and *MAOop* in pET-23a | Matsumura et al., Sci Rep., 2018 |
| pACYC-3MT-DDC | *6OMT*, *CNMT*, *DODC*, and *4'OMT* in pACYC184 | Matsumura et al., Sci Rep., 2018 |
| pAO815- *EcBBE- EcCYP719A5- EcCYP719A2* | *EcBBE, EcCYP719A5 and EcCYP719A2* in pAO815 | Hori et al., Sci Rep., 2016 |

op: codon optimized
