# Supplementary material for: Establishment of a co-culture system using Escherichia coli and Pichia pastoris (Komagataella phaffii) for valuable alkaloid production
Source: Microb Cell Fact. 2021 Oct 18;20:200. doi: 10.1186/s12934-021-01687-z (PMC8522034; doi:10.1186/s12934-021-01687-z)
Supplement: Supplementary file 2 — Additional file 2: Fig. S1. Detection of (S)-stylopine in various media. Single-ion chromatogram of (S)-stylopine in P. pastoris cells grown in each medium and authentic standard. N.D.; not detected. Fig. S2 (S)-Reticuline production in the co-culture of E. coli and P. pastoris. Cells were cultured as per methods described in the Fig. 4 legend. (S)-Reticuline in the cells (a) and medium (b) were detected and quantified. Results indicate mean ± standard deviation of triplicate experiments. Fig. S3 Growth and cell density of E. coli and P. pastoris in co-culture system. An initial inoculation ratio of E. coli and P. pastoris cells was 0.3:0.1. Growth was evaluated by measuring the optical density at 600 nm (a). The number of E. coli cells (b) and P. pastoris cells (c) were counted using a bacteria counting chamber and a microscope. Results indicate mean ± standard deviation of technical triplicates. [file 12934_2021_1687_MOESM2_ESM.pptx]

## Slide 1
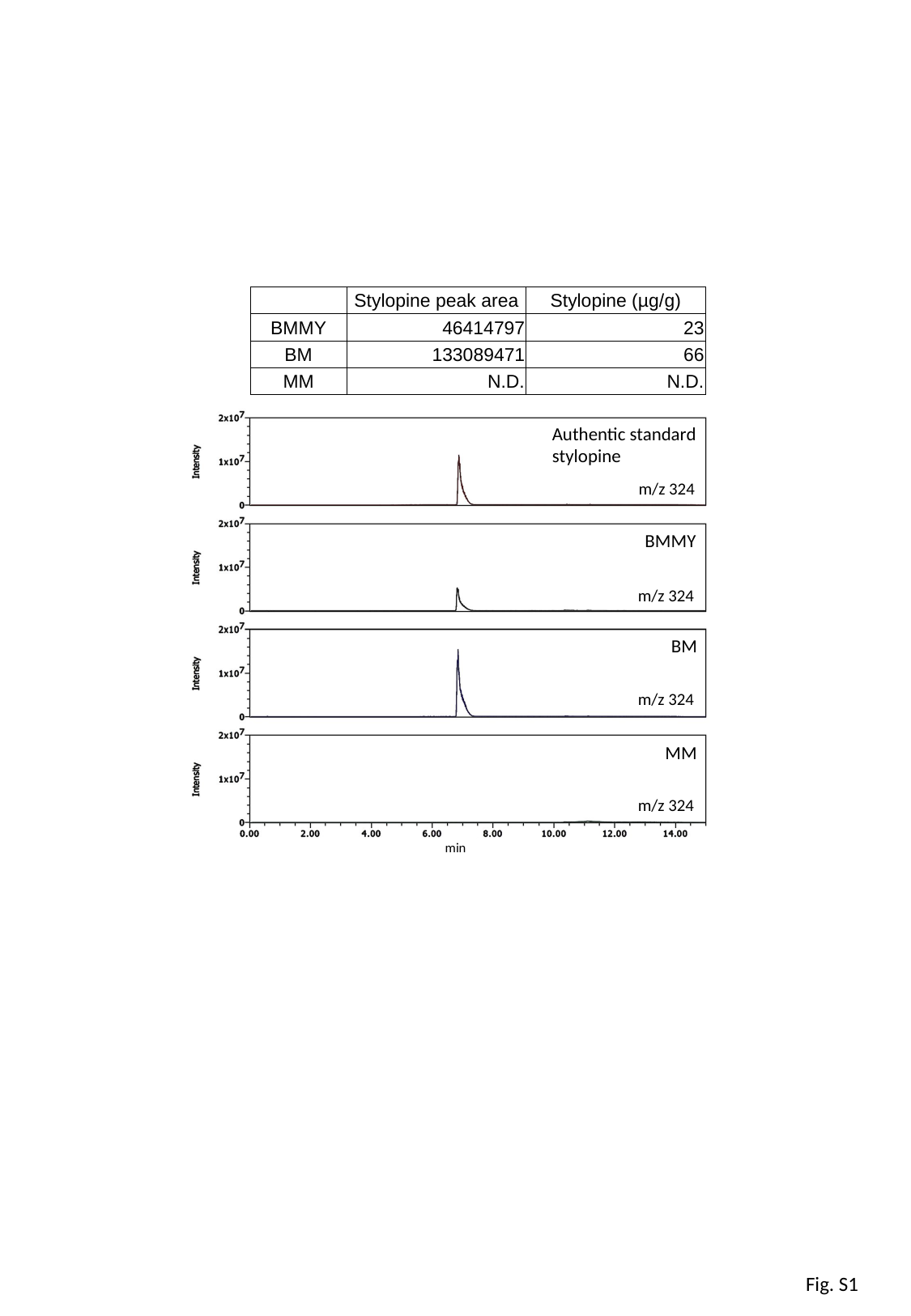

| | Stylopine peak area | Stylopine (µg/g) |
| --- | --- | --- |
| BMMY | 46414797 | 23 |
| BM | 133089471 | 66 |
| MM | N.D. | N.D. |
Authentic standard
stylopine
m/z 324
BMMY
m/z 324
BM
m/z 324
MM
m/z 324
min
Fig. S1

## Slide 2
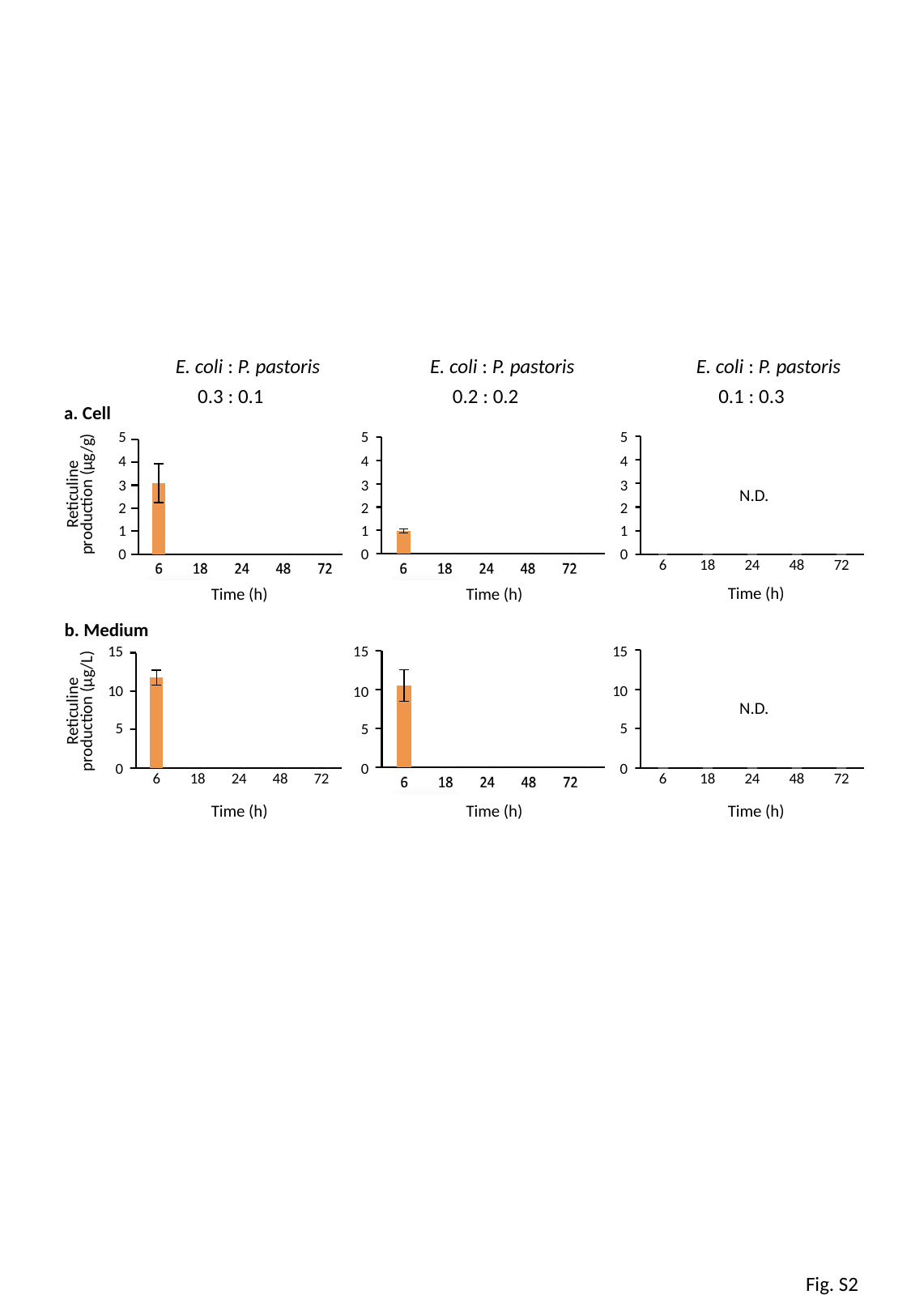

E. coli : P. pastoris
E. coli : P. pastoris
E. coli : P. pastoris
0.3 : 0.1
0.2 : 0.2
0.1 : 0.3
a. Cell
### Chart
| Category | |
|---|---|
| 6 | 0.0 |
| 18 | 0.0 |
| 24 | 0.0 |
| 48 | 0.0 |
| 72 | 0.0 |
### Chart
| Category | |
|---|---|
| 6 | 0.9741249867733334 |
| 18 | 0.0 |
| 24 | 0.0 |
| 48 | 0.0 |
| 72 | 0.0 |
### Chart
| Category | |
|---|---|
| 6 | 3.0911208711644442 |
| 18 | 0.0 |
| 24 | 0.0 |
| 48 | 0.0 |
| 72 | 0.0 |5
5
5
4
4
4
3
3
3
Reticuline
production (µg/g)
N.D.
2
2
2
1
1
1
0
0
0
 Time (h)
 Time (h)
 Time (h)
### Chart
| Category | |
|---|---|
| 6 | 0.0 |
| 18 | 0.0 |
| 24 | 0.0 |
| 48 | 0.0 |
| 72 | 0.0 |
### Chart
| Category | |
|---|---|
| 6 | 10.491506597037036 |
| 18 | 0.0 |
| 24 | 0.0 |
| 48 | 0.0 |
| 72 | 0.0 |b. Medium
### Chart
| Category | |
|---|---|
| 6 | 11.775703669135803 |
| 18 | 0.0 |
| 24 | 0.0 |
| 48 | 0.0 |
| 72 | 0.0 |
15
15
15
10
10
10
Reticuline
production (µg/L)
N.D.
5
5
5
0
0
0
 Time (h)
 Time (h)
 Time (h)
Fig. S2

## Slide 3
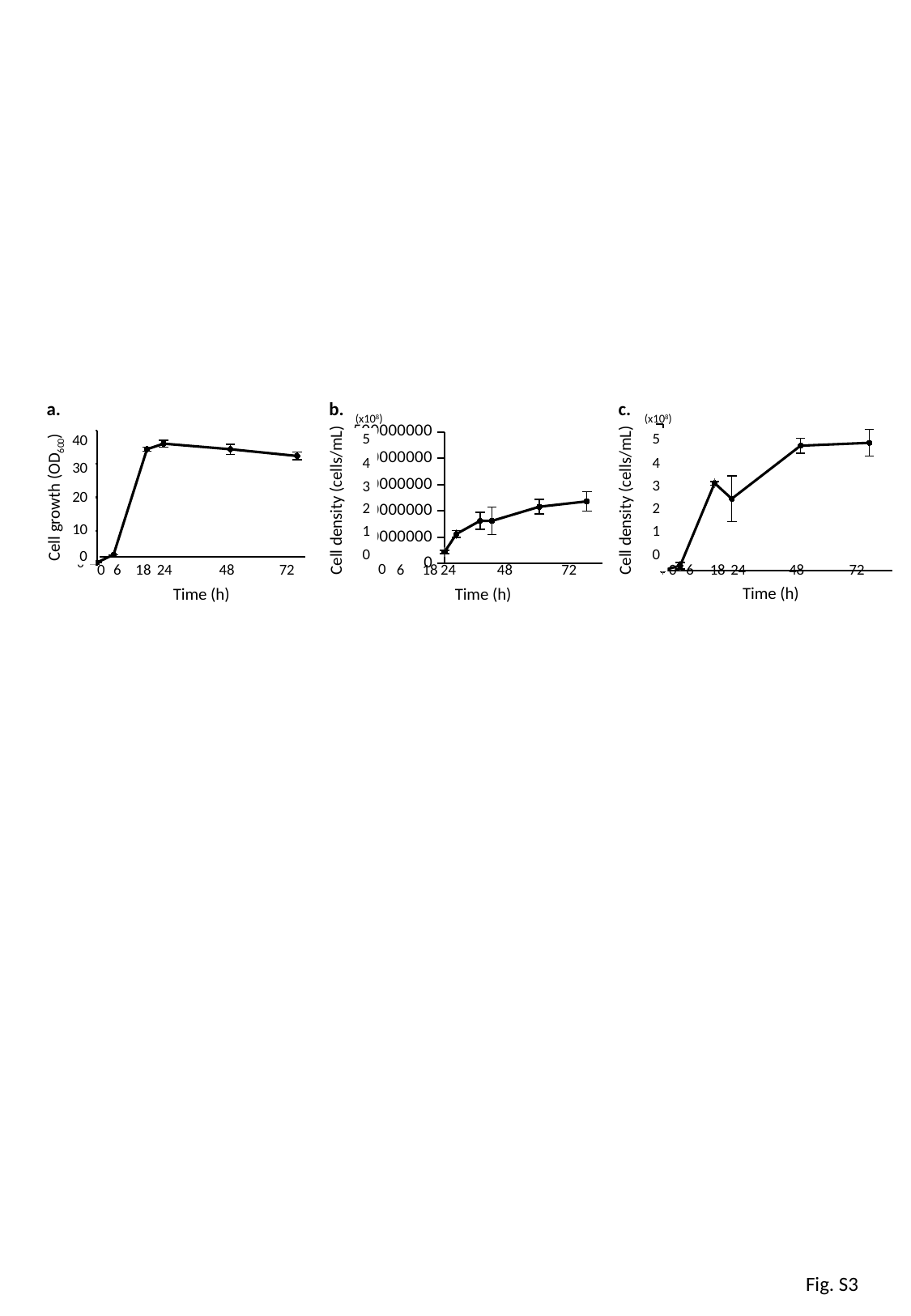

a.
b.
c.
(x108)
(x108)
### Chart
| Category | |
|---|---|
### Chart
| Category | |
|---|---|
### Chart
| Category | |
|---|---|5
5
40
4
4
30
3
3
20
Cell growth (OD600)
Cell density (cells/mL)
Cell density (cells/mL)
2
2
10
1
1
0
0
0
0
0
6
18
24
48
72
6
18
24
48
72
6
18
24
48
72
0
 Time (h)
 Time (h)
 Time (h)
Fig. S3
